# On the Effects of Package on the PMUTs Performances—Multiphysics Model and Frequency Analyses

**DOI:** 10.3390/mi11030307

**Published:** 2020-03-14

**Authors:** Gianluca Massimino, Alessandro Colombo, Raffaele Ardito, Fabio Quaglia, Alberto Corigliano

**Affiliations:** 1 Department of Civil and Environmental Engineering, Politecnico di Milano, 20133 Milan, Italy; gianluca.massimino@polimi.it (G.M.); raffaele.ardito@polimi.it (R.A.); 2 Analog, MEMS & Sensors Group, ST Microelectronics, Via Tolomeo 1, 20010 Cornaredo, Italy; fabio.quaglia@st.com

**Keywords:** acoustic-structure interaction, array, flexible package, multiphysics modelling, piezoelectric tranducers, ultrasound

## Abstract

This paper deals with a multiphysics numerical modelling via finite element method (FEM) of an air-coupled array piezoelectric micromachined ultrasonic transducers (PMUTs). The proposed numerical model is fully 3D with the following features: the presence of the fabrication induced residual stresses, which determine a geometrically non-linear initial deformed configuration of the diaphragms and a remarkable shift of the fundamental frequency; the multiple coupling between different physics, namely electro-mechanical-coupling for the piezo-electric model, acoustic-structure interaction at the acoustic-structure interface and pressure acoustics in the surrounding air. The model takes into account the complete set of PMUTs belonging to the silicon die in a 4 × 4 array configuration and the protective package, as well. The results have been validated by experimental data, in terms of initial static pre-deflected configuration of the diaphragms and frequency response function of the PMUT. The numerical procedure was applied, to analyze different package configurations of the device, to study the influence of the holes on the acoustic transmission in terms of SPL and propagation pattern and consequently extract a set of design guidelines.

## 1. Introduction

The application of piezoelectric materials in “smart” microsystems [1] is continuously increasing, with different possible uses of both “direct” (conversion of mechanical energy into electric energy) and “converse” effect [2]. The latter is applied for actuating purposes, for example, in the case of micropumps [3,4,5]; “converse” effect is now widely used for energy harvesting, namely for obtaining an electric power by exploiting some freely available mechanical energy [6,7].

Piezoelectric micromachined ultrasonic transducers (PMUTs) are layered plates with a piezoelectric active layer used for emitting and receiving ultrasonic waves [8,9,10]. Nowadays, they are used for many applications like range-finding [11,12,13], gesture recognition [14], finger-printing recognition [15], exploiting the in-air propagation [16], ultrasound imaging [17,18] and sonography [18] in which the in-water propagation is involved [19]. Further applications are represented by non-destructive testing, velocity sensing [20].

**Figure 1:**
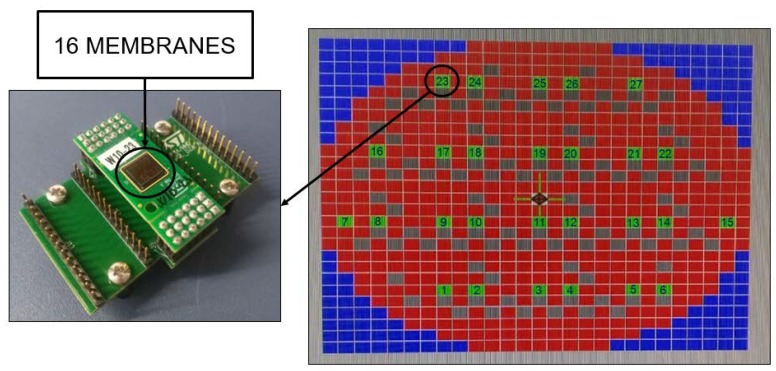
View of the 4 × 4 array of circular transducers (**left**); position of the sample on the wafer map (**right**).

**Figure 2:**
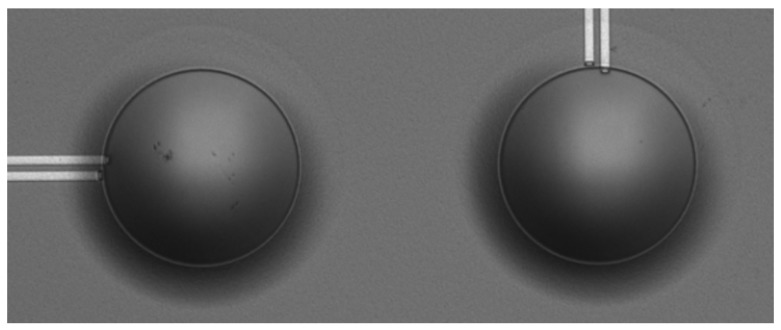
Optical microscope image of two piezoelectric micromachined ultrasonic transducers (PMUTs).

This paper builds upon a previous work [21] and deals with a fully 3D modelling of a 4 × 4 PMUTs array configuration presented in Figure 1 and Figure 2.

This is perfectly suited to simulate the presence of the flexible protecting package of any possible shape. Moreover, the model is characterized by the acoustic-structure interaction enforced at each acoustic-structure interface, so the interaction does not involve only the vibrating diaphragms but the package as well [22].

The primary aim of this work is to investigate the influence of the flexible protective package on the acoustic performances of the PMUT devices. As a matter of fact, different package shapes at different performing frequencies will be studied in terms of Sound-Pressure-Level (SPL) maps and polar plots. Moreover, the effects of the package vibration modes on the pressure radiation lobes will be illustrated.

The paper is organized as follows. The second section describes the multiphysics finite element (FE) modelling of both the electro-mechanical and the acoustic behavior of the transducers, account taken of the multiple interactions among the involved physics [23,24]. The model gives a realistic estimation of the pre-deflected configuration, due to the residual stress state and of the fundamental frequency.

In the third section, the corresponding numerical results are reported showing a good agreement with the experimental ones. In section four, the stand-alone performances of the transducer in the transmission phase, for the packaged and unpackaged device, are shown. Furthermore, the 4 × 4 array of PMUTs is considered and several package solutions are analyzed to define advantages and drawbacks for each simulated flexible protective cap shapes.

Section 5 is devoted to conclusions. Closing remarks are reported on the modelling technique and the different pressure scenarios related to the adopted package. Finally, possible future developments are presented.

## 2. Numerical Modelling for the Single Transducer

The finite element model was developed in ANSYS 17.2. In the numerical model the electro-mechanical-acoustic (EMA) coupling was taken into account, for a fully 3D modelling of the device and the surrounding air. The thermo-viscous acoustics model for the air near to the vibrating diaphragms, which was considered in Reference [25], is here neglected since the primary focus is on the pressure and Sound-Pressure-Level (SPL) far-field responses.

**Figure 3:**
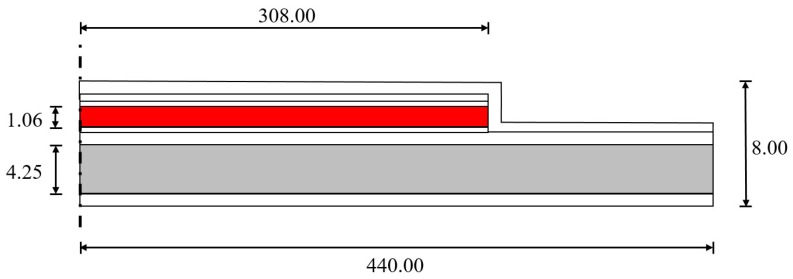
PMUT layered configuration with PZT, Si dimensions and overall thickness in μm: PZT in red, Si structural layer in grey, other layers in white.

Each circular transducer has radius of 440 μm and an overall thickness equal to 8 μm, such that the diameter/thickness ratio is 110. The structural layer is made of silicon with thickness equal to 4.25 μm. A thin piezoelectric film, made of lead zirconate titanate (PZT) [26] is deposited via sol–gel technique, in hat configuration on the structural plate. The PZT layer has a thickness of 1.06 μm and is placed in circular hat configuration with radius of 308 μm, coaxial with the diaphragm. The typical layered configuration is shown in Figure 3 in which a cross-section, in the meridian plane of the geometry, is reported.

The piezo-plate belongs to the silicon die. Beneath the PMUT an air-filled closed cylindrical cavity with height of 400 μm and the same radius of the upper diaphragm is present.

**Figure 4:**
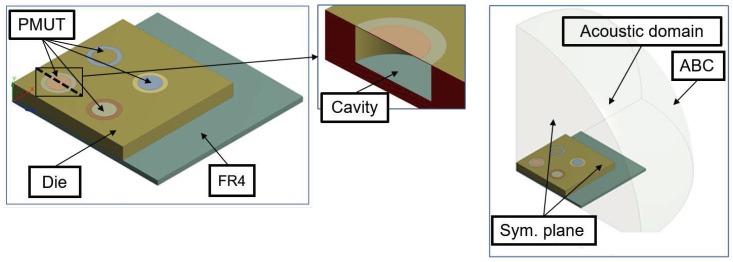
Geometry for the electro-mechanical-acoustic model: a quarter of 4 × 4 array of PMUTs (**left**), cross section of the transducers with the cavity (**center**), solid and acoustic domain (**right**).

**Figure 5:**
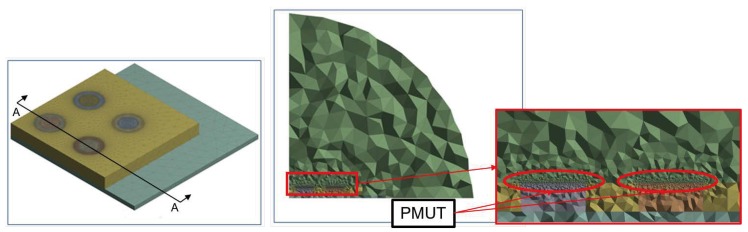
Mesh details: solid domain (**left**), vertical cross-section along A-A of the solid and acoustic domains (**center**), structure-acoustic interface (**right**).

The die has dimensions equal to 7.2 × 7.2 × 0.4 mm${}^{3}$ and it is glued onto the printed circuit board (PCB) made of FR4 whose bottom face is modelled as a fixed constraint and with dimensions equal to 10.00 × 10.00 × 0.15 mm${}^{3}$. The symmetry properties of the device is exploited to reduce the computational burden, modelling just a quarter of the device with 2 × 2 transducers and applying Symmetric Boundary Condition on the two vertical planes. To simulate the radiation into an infinite medium, the absorption condition at the air quarter of sphere boundary is enforced. On the horizontal surface boundary of the acoustic domain, on the contrary, a hard wall condition is imposed. The model, without the protecting cap, is presented in Figure 4 and Figure 5, in which the geometry and the mesh details for the different physical domains are shown.

The physics describing the simulated phenomenon are: elastodynamics in the linear elastic layered system, silicon die and PCB, electrostatics in the piezoelectric layer with enforced linear stress-charge law and pressure acoustics in the air cavity and in the quarter of sphere air domain with radius equal to 3λ=10.302 mm, where $\lambda =\nu_{s}/f_{0}=3434$ μm is the wavelength, $\nu_{s}=343$ m/s is the speed of sound in the air, computed at the linearization reference state characterized by $T_{ref}$ = 293.15 K and $P_{ref}$ = 1 atm and $f_{0}$ = 100 kHz is the performing frequency of the system, that coincides with the plate fundamental frequency in order to maximize the acoustic efficiency while the in-air standard SPL reference pressure $p_{0}$ = 20.4 μPa is used to compute the SPL maps and polar plots.

The FR4 board and the silicon die were discretized with SOLID226 linear tetrahedrons. The mesh of the plate was obtained by means of the swept mesh generation technique with solid-like SOLSH190 linear wedge elements for all the layers but the PZT. The piezoelectric domain was modelled by means of three SOLID226 piezoelectric linear wedge elements through the thickness. In the silicon structural layer, five wedge elements through the thickness were used. Conversely, at least one element through the thickness was inserted in the remaining diaphragm domain, adopting a minimum element size equal to the smallest thickness while the maximum size was set equal to the thickness of silicon die.

**Figure 6:**
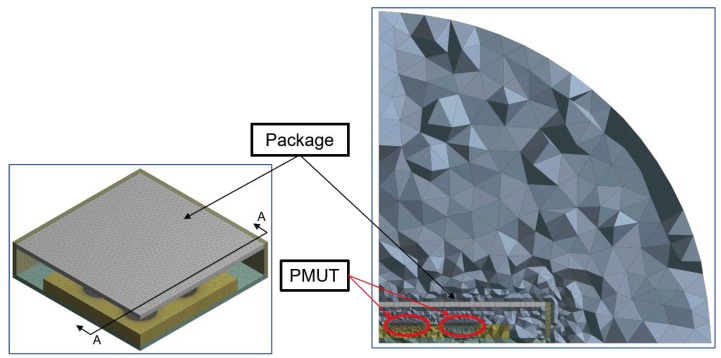
Mesh details: packaged transducer with no hole (**left**), vertical cross-section along A-A of the solid and acoustic domains (**right**).

The adopted model to study the pressure propagation in the presence of the protecting cap for the transducer, is shown in Figure 6. Herein, the package is constituted of brass which has thickness of 150 μm. In order to study the influence on the pressure radiation pattern, related to the activated vibration modes of the protecting structure, it is discretized as a linear elastic material through SOLSH190 linear wedge elements, with one element through the thickness. Hence, the Fluid-Structure Interaction (FSI), in the form of Acoustic-Structure Interaction (ASI) is enforced not only at the vibrating diaphragms structural-acoustic interfaces but at the package wet surface as well. It is worth noting that, the linear elastic package modelling and the adopted ASI at the package boundary are needed, in order to correctly simulate the pressure propagation in the surrounding air. Otherwise, in the case of package without any vent, presented in Figure 6, no acoustic radiation would occur.

**Figure 7:**
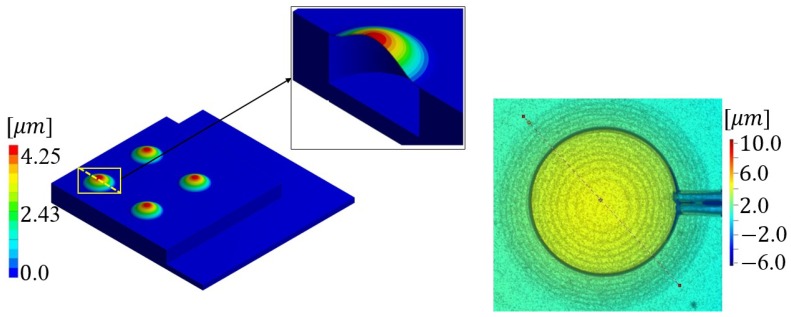
Initial static configuration due to fabrication residual stresses: numerically computed by means of ANSYS 17.2 (μm) (**left**); experimental measurement by means of Polytec MSA-500, top view (**right**).

**Figure 8:**
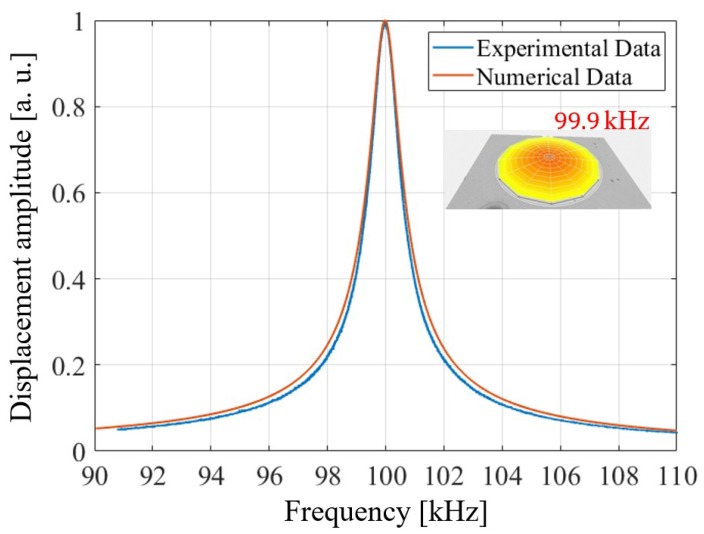
Comparison between experimental and numerically computed transversal normalized displacement frequency amplitude spectrum at the center of the PMUT by means of ANSYS 17.2 with experimentally computed fundamental mode shape at 99.9 kHz (**top right**).

The fabrication induced residual stresses are related to thermally induced deformations, during the fabrication process. The thin plate configuration of the transducer implies the out of plane stress is negligible with respect to the other stresses. Moreover, the measured initial deformation is characterized by the axial symmetry, the epitaxial fabrication process and the sol-gel technique, used to build the PZT, are isotropic with respect to the in-plane directions. Hence, they are taken into account through an amount of pre-stress isotropically acting in the plane of each layer, so that the cross section is characterized by tensile and compressive initial stresses. They play an important role in the initial deformation and on the geometric stiffness of the structure in the large displacement formulation and their estimated values, together with the isotropic in-plane approximation, provide very good results, as show in Figure 7 and Figure 8.

The quality factor of the system, related to the fundamental mode, was measured by means of a free vibration decay test and corresponds to $Q_{tot}=80$. It depends on several sources of energy losses, namely: structural losses $Q_{struct}$, which model the effects of the thermoelastic, support, surface layer losses, and fluid losses $Q_{fluid}$, related to the energy radiation into an infinite medium constituted of air. In the EMA frequency analysis of the device, considering the adopted absorbing condition at the acoustic boundary, the overall structural damping was introduced by means of an enforced damping ratio, equal to $\zeta =1/2Q_{struct}$ in the dynamic equations of motion, where a numerical $Q_{struct}=100$ was imposed to obtain the acceptable match between the numerical and the known experimental $Q_{tot}$. Accordingly, the numerical $Q_{fluid}=258$ was computed subtracting the inverse of $Q_{struct}$ to the inverse of the $Q_{tot}$ and extracting the inverse of the result (as common in parallel impedance reduction) [27].

In the pressure acoustic hemisphere, the Helmholtz equation was solved, adopting a discretization constituted of FLUID30 linear tetrahedral elements with maximum element size equal to λ/6. The acoustic-structure interaction is enforced by means of the continuity of the normal stresses and of the normal accelerations at the wet boundaries. Moreover, Absorbing Boundary Condition (ABC) on the quarter of sphere pressure acoustic boundary was modelled by means of FLUID130 linear triangular elements.

The far-field approximation is valid above the complex near-field domain and far from the emitting source. Considering a circular vibrating transducer, it holds in points placed farther than the Rayleigh distance $D_{R}$, and that satisfy the inequality 2r/d>>1, where *r* is the distance between the point and the transducer center, and *d* is the transducer diameter. In order to demonstrate the complex near-field is fully enclosed in the computational acoustic domain, the smallest distance between a transducer and the ABC acoustic boundary can be estimated as $r_{min}=3\lambda -\sqrt{\left(x_{0}^{2}+y_{0}^{2}\right)}=6.95$ mm, where $\left(x_{0},y_{0}\right)$ are the coordinates of the center of a PMUT placed at a die corner. The Rayleigh distance is equal to $D_{R}=\pi d^{2}/4\lambda =S/\lambda =177.1$ μm, where d=880 μm is the PMUT diameter and $S=\pi d^{2}/4=0.608$ mm${}^{2}$ is the PMUT area [28]. Therefore, considering the points at the ABC acoustic boundary, the requirements are largely fulfilled. As a consequence, the far-field quantities are correctly evaluated by means of the Helmholtz-Kirchhoff integral technique, based on the acoustic properties of air, considering the ABC acoustic boundary as the reference integration surface [28,29].

The electro-structural-acoustic response is computed at two different performing frequencies by means of a sequence of simulations: (i) a geometrically non-linear static analysis was performed to compute the pre-deflected configuration induced by the fabrication residual stresses, (ii) an electro-mechanical static analysis was performed to compute the starting performing configuration, considering two bias voltage levels of 3 V and 10 V, (iii) an electro-mechanical eigenfrequency analysis, around the previously computed performing starting deformed configuration, was carried out to evaluate the fundamental frequency of the device at the two bias levels, (iv) an electro-mechanical-acoustic frequency analysis at the performing diaphragms fundamental frequency was performed, around the stressed deformed starting configuration, to compare the solutions for a harmonic voltage perturbation with amplitude of 3 V account taken of the constant bias voltage. The goal was to investigate the performances of the system in the transmission phase (TX) at two different performing PMUTs fundamental frequencies, under the influence of the starting configuration due the combination of the pre-stresses and the bias voltage and of the adopted package geometry.

## 3. Numerical Results and Experimental Validation

In this section, the comparison between the experimental and numerical results of the first study, described in the previous section, are shown (Figure 7). To compute the starting configuration of the transducers, which is characterized by a high aspect ratio (diameter/thickness) of 110, a large displacements static analysis was carried out under the influence of the residual stress state. The numerically computed displacement of the diaphragm is characterized by a maximum value of 4.25 μm, which occurs at the center point, in the vertical direction. The corresponding experimental measurement was executed via the white-light interferometry by means of the Polytec MSA-500 microsystem analyzer. The related displacement contour plot map together with the plate profile along a diameter show a maximum value at the center equal to 4.2 μm, as reported in Figure 7 in which the PZT hat displacements are in the range (3.6 μm, 4.2 μm), of the corresponding yellow color in the legend beside.

In addition to the initial deformed shape, the residual stresses play the most important role in the geometric stiffness of the diaphragm. Indeed, the initial state of stress significantly changes the fundamental frequency of the PMUT. It is worth noting that the diaphragm without the residual stresses presents a theoretical fundamental eigenfrequency of 111.55 kHz; taking into account the contribution to the geometric stiffness due to the pre-stress state, it decreases to 99.9 kHz and is correctly captured by the numerical model that predicts 100.0 kHz. To identify the shift, the electro-mechanical-acoustic frequency sweep analysis was performed with a harmonic voltage excitation with amplitude of 3 V and zero DC voltage bias, around the fundamental frequency. The result of the study in terms of transversal displacement amplitude of the center of the plate, is reported in Figure 8 together with the experimental data, obtained through the Polytec MSA-500 laser-doppler vibrometer frequency sweep test with the excitation of 1 V amplitude and zero DC voltage bias. The simulation methodology was fully validated with acoustic measurements in the authors published work [8]. In the following package case studies, the presence of protecting structure was modelled as an added linear elastic material of the system. The features of the model are exactly the same as in the previous paper, the only difference being the presence of another elastic part.

## 4. Analyses in Presence of Package

In this section, different package configurations are presented with the effects on the transducer performance in terms of acoustic intensity and wave propagation pattern.

### 4.1. Stand-Alone Device vs. Packaged Device

The effect of the protecting cap on the transmission performances of the transducer is studied through the numerical comparison between the stand-alone device and the packaged.

The adopted acoustic finite element modelling of the surrounding air allows one to evaluate the acoustic efficiency [30] of the PMUT in the air domain in terms of Sound Pressure Level (SPL) at the distance of 3.5 cm from the center of the die, along the acoustic vertical axis of the device. Furthermore, the model was used to get the pressure propagation map and the SPL radiation beam pattern in the polar plot, related to the analyzed package geometry and dynamic behavior. Hence, it represents a powerful tool to study directivity and beam-forming problems involving the whole vibrating system.

To this aim, the pressure field was computed in every point of the computational air domain and outside of it by means of the Kirchhoff- Helmholtz integral technique (the so called far-field calculation technique), based on the pressure values and gradients evaluated on an integration surface which encompass the entire device.

**Figure 9:**
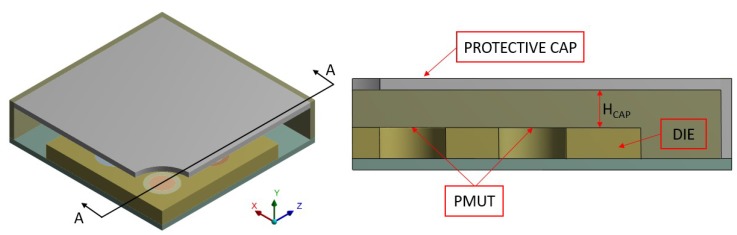
Package configuration: geometry quarter (**left**), cross-section A-A and parameters (**right**).

The parameters which define the protecting cap are illustrated in Figure 9. The distance $H_{cap}$, from the top surface of the die to the bottom surface of the cap, is equal to 500 μm. In the presence of the holed package configuration the parameter $R_{hole}$ refers to the radius of the vent and it is expressed in terms of the PMUT radius $R_{PMUT}$.

**Figure 10:**
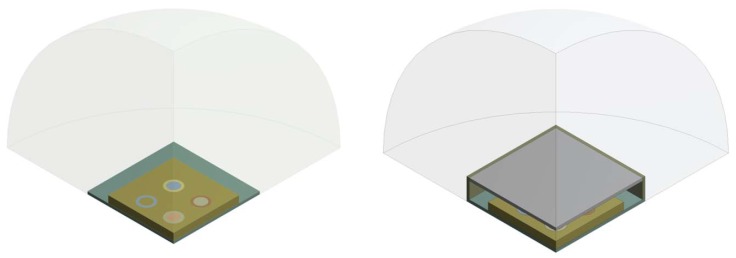
Analyzed limit situations: stand-alone device (**left**), packaged device (**right**).

The two limit situations, illustrated in Figure 10, were simulated, in which the transducer is not protected by any system and in the presence of the package with no vent ($R_{hole}=0$ μm), characterizing the maximum protection against any external agent.

**Figure 11:**
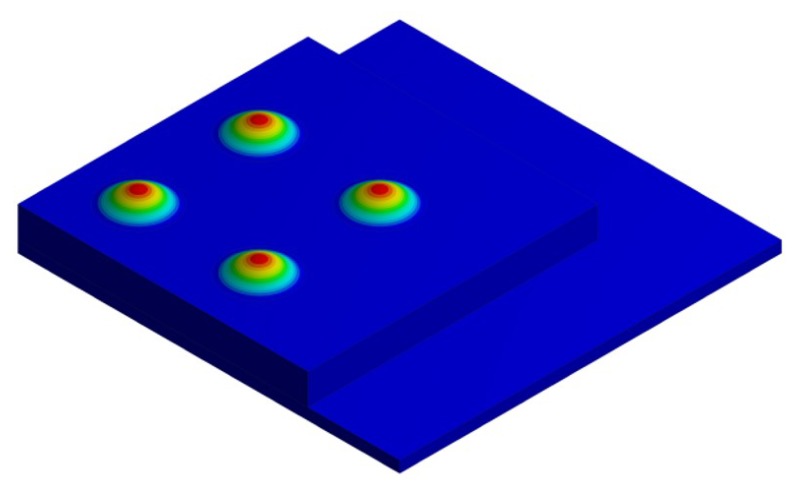
Fundamental electro-mechanical displacement mode shape of the stand-alone device 93.0 kHz (3 V bias), 85.5 kHz (10 V bias).

**Figure 12:**
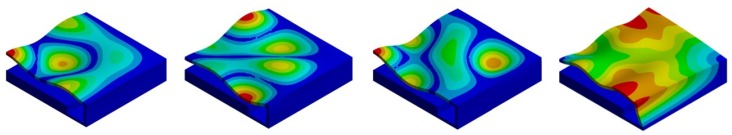
Four package displacement eigenmodes around the fundamental frequency: 6th mode shape at 81.46 kHz (**left**), 7th mode shape at 104.18 kHz (**center-left**), 8th mode shape at 104.45 kHz (**center-right**) 16th mode shape at 158.110 kHz (**right**).

To investigate the dynamic behavior of the device in the two cases, a non-linear electromechanical-eigenfrequency analysis was performed, taking into account the presence of the residual stress state on the geometric stiffness. The results of the study are shown in the following two figures in terms of displacement eigenmode of the stand-alone and the packaged device; the former (Figure 11) corresponds to the performing fundamental frequency of the designed PMUTs taking into account the residual stresses and a constant bias voltage of 3 V and 10 V, while the latter (Figure 12) shows the related package displacement eigenmodes, around the fundamental frequencies of the transducer for the two considered voltage bias.

Due to the presence of the protective package, the acoustic waves propagate inside the acoustic domain through the cap vibration. Therefore, it is useful to investigate the package eigefrequencies and the associated mode shapes to detect which ones are involved in the acoustic propagation, characterizing the device acoustic performances in terms of acoustic intensity and directivity. In Figure 12 the attention is focused on the four package displacement eigenmodes, around the performing fundamental frequencies of the transducers 85.5 kHz (10 V bias) and 93.0 kHz (3 V bias) and together with the first package eigenmode with deformed walls. The shown package eigenfrequencies are not significantly affected by the applied static bais voltage. To investigate the SPL maps, the response was obtained in the frequency domain, considering all the PMUTs actuated in parallel with a harmonic voltage excitation of 3V amplitude and two different bias voltage levels, respectively of 10 V and 3 V, at the corresponding fundamental frequencies of the plates equal to 85.5 kHz (applying 10 V bias) and 93.0 kHz (applying 3 V bias).

**Figure 13:**
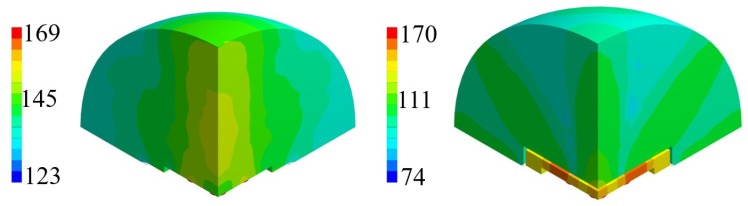
SPL contour plot (dB) at the performing fundamental frequency of 85.5 kHz (10 V bias): unpackaged device (**left**), packaged device (**right**).

**Figure 14:**
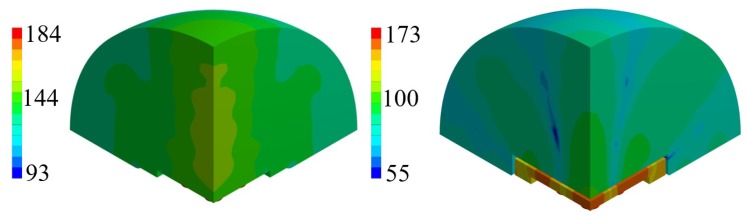
SPL contour plot (dB) at the performing fundamental frequency of 93.0 kHz (3 V bias): unpackaged device (**left**), packaged device (**right**).

The comparison between unpackaged and package devices, in terms of numerically computed SPL contour plot, are presented in Figure 13 and Figure 14 for the cases of constant bias voltage of 10 V and 3 V.

**Figure 15:**
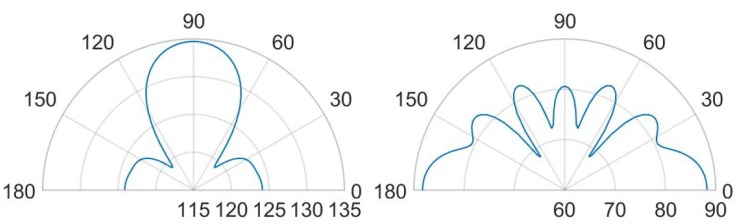
SPL polar plot (dB) at the performing fundamental frequency of 85.5 kHz (10 V bias), at 3.5 cm in the y(90${}^{\circ }$)-z(0${}^{\circ }$) plane: unpackaged device (**left**), packaged device (**right**).

**Figure 16:**
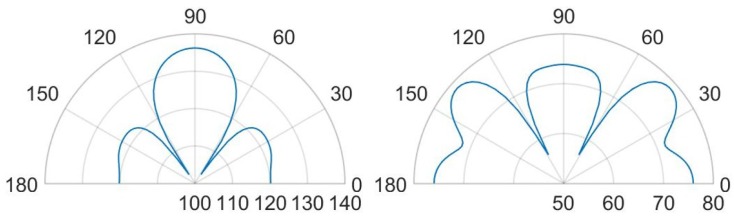
SPL polar plot (dB) at the performing fundamental frequency of 93.0 kHz (3 V bias), at 3.5 cm in the y(90${}^{\circ }$)-z(0${}^{\circ }$) plane: unpackaged device (**left**), packaged device (**right**).

It is worth noting that, for both the analyzed scenarios, the maximum value in the SPL occurs in each cavity below the transducers. Moreover, in presence of packaging, there are high values of SPL in the acoustic domain in between the silicon die and the protective cap, which cause several reflections of the acoustic waves inside the device and the package vibration. Furthermore, the presence of the cap without holes reduces the maximum value of SPL along the vertical acoustic axis of the system passing through the center of the device, as it is reported, for both the actuation frequencies, in Figure 15 and Figure 16 as well. In these figures the SPL polar plots are sketched at a distance of 3.5 cm from the center of the die and in the vertical plane y-z passing through the center of the system at the analysis frequencies.

Considering the simulated package configuration, a vertical lobe and two side lobes of propagation, at 47${}^{\circ }$ from the vertical axis of 90${}^{\circ }$ in the y-z plane, are visible together with the horizontal one at 0–180${}^{\circ }$, due to the package walls vibration.

Concerning the unpackaged system, it exhibits the maximum value of SPL along the vertical direction of 90${}^{\circ }$ with the same intensity for the two analyzed fundamental frequencies [31]. This is basically in accordance with the lack of the protective cap, which reflects the acoustic waves inside the device and determines the wave propagation in the surrounding air according with its own excited vibration modes. In the cap-free configuration one can notice that the vertical lobe shape has no significant variation at the two frequencies of 85.5 kHz and 93.0 kHz. There are slightly different patterns and values at 30${}^{\circ }$ and the horizontal directions, due to the different initial deformed configurations, under the bias static voltage, around which the vibration starts. the applied bias voltage contracts the initial deformation due to the residual stresses, hence the most deformed configuration is associated with the lowest bias voltage of 3V and fundamental frequency of 93.0 kHz. Consequently, at 93.0 kHz, the system tends to spread the acoustic energy on spherical wavefronts along the horizontal direction and the transmission becomes more intense approaching the vertical direction, due to the waves composition coming from all the involved vibrating PMUTs.

Considering the packaged system, the acoustic intensity in the surrounding air is lower with respect to the unpackaged device, due to the obstructive effect induced by the protective cap without any vent. Further considerations about the wave propagation pattern are based on the SPL polar plots. As a matter of facts, Figure 16 predicts a flat main vertical lobe between 70${}^{\circ }$ and 110${}^{\circ }$ of 74 dB, at the frequency of 93.0 kHz. While, Figure 15 shows three separate narrow lobes of propagations around the same interval of polar angle with local maxima of about 80 dB for the vertical one and 83 dB for the specular side ones.

Horizontal propagation of the acoustic waves characterizes both the analyzed cases, with higher values of SPL for the unpackaged system, due to the fact that there are no obstacle to the transmission, as the package walls are. The packaged system, indeed, is characterized by the SPL values of 88 dB along the direction of 0–180${}^{\circ }$ at the frequency of 85.5 kHz, while at 93.0 kHz, the SPL value along the horizontal direction is equal to 76 dB while the maximum intensity of 78 dB is reached along the directions of 43${}^{\circ }$ and 137${}^{\circ }$.

### 4.2. Simulations for Different Package Geometries

**Figure 17:**
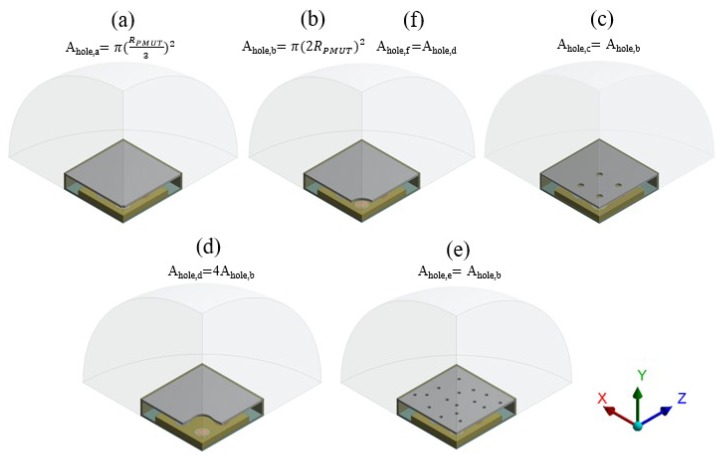
Different cap geometries as described in the main text.

Going further, several simulations were performed considering devices with different kind of protective caps. To investigate the propagation patterns in the far field. Six new configurations of the protective cap are sketched in Figure 17: (a) central hole of radius equal to $R_{hole}=R_{PMUT}/3$ and $A_{hole,a}=\pi \cdot {\left(\frac{R_{PMUT}}{3}\right)}^{2}$; (b) central hole of radius equal to $R_{hole}=2R_{PMUT}$ and $A_{hole,b}=\pi \cdot {\left(2R_{PMUT}\right)}^{2}$; (c) holes placed at the center of each transducer, with radius equal to $R_{hole}=R_{PMUT}/2$ and total holed surface equal to $A_{hole,c}=A_{hole,b}$; (d) hole at the center of the package, suggested by the SPL shape on the outer cap surface in the case of the previously analyzed no-hole situation (see Figure 10, right picture) and total holed surface $A_{hole,d}=4A_{hole,b}$; (e) random holes of radius equal to $R_{hole}=R_{PMUT}/4$ and total holed surface equal to $A_{hole,e}=A_{hole,b}=A_{hole,c}$; (f) central hole, as case (b) but with different radius equal to $R_{hole}=\sqrt{A_{hole,d}/\pi }$, where $A_{hole,d}$ is the holed area in the case (d) that implies $R_{hole}=4R_{PMUT}$, two times greater than case (b) and holed area $A_{hole,f}=A_{hole,d}$. The SPL polar plots—at the reference distance of 3.5 cm from the center of the die, in the vertical plane y-z passing through the center of the system—were computed at the performing fundamental frequencies of the transducers and they are showed in the following figures for the case (a–e). Case (f) will be described in the next section in which several comments on the acoustic transmission efficiency, considering the comparison plots between two analyzed situations, will be drawn.

**Figure 18:**
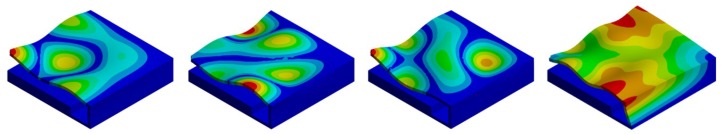
Four package modes for case (a): 6th mode shape at 81.32 kHz (**left**), 7th mode shape at 104.13 kHz (**center-left**), 8th mode shape at 104.29 kHz (**center-right**), 16th mode shape at 157.53 kHz (**right**).

**Figure 19:**
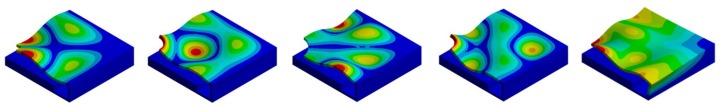
Five package modes for case (b): 5th mode shape at 78.86 (**left**), 6th mode shape at 85.02 kHz (**center-left**), 7th mode shape at 103.45 kHz (**center**), 8th mode shape at 110.32 kHz (**center-right**), 13th mode shape at 136.14 kHz (**right**)).

**Figure 20:**
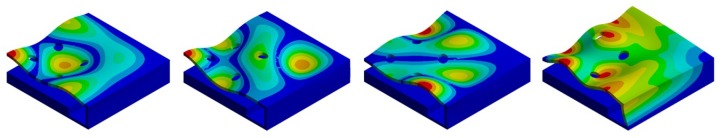
Four package modes for case (c): 6th mode shape at 80.18 kHz (**left**), 7th mode shape at 102.66 kHz (**center-left**), 8th mode shape at 102.66 kHz (**center-right**), 16th mode shape at 151.01 kHz (**right**).

**Figure 21:**
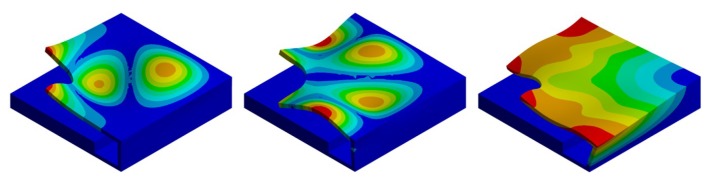
Three package modes for case (d): 6th mode shape at 91.53 kHz (**left**), 7th mode shape at 101.82 kHz (**center**), 8th mode shape at 108.23 kHz (**right**).

**Figure 22:**
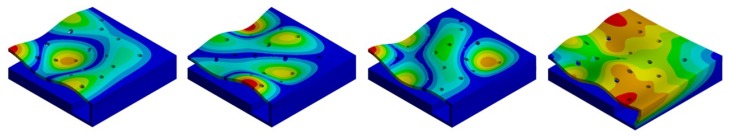
Four package modes for case (e): 6th mode shape at 80.13 kHz (**left**), 7th mode shape at 102.43 kHz (**center-left**), 8th mode shape at 102.81 kHz (**center-right**), 16th mode shape at 154.38 kHz (**right**).

To identify the modes of vibration involved in pressure waves propagation, an eigenfrequency analysis was performed for the five cap configurations, taken into account the residual stress state and the applied static bias voltages; the results in terms of eigenmode shapes, around the transducers performing fundamental frequencies of 85.5 kHz (10 V bias) and 93.0 (3 V bias), together with the package eigenmode with deformed walls, are shown in Figure 18, Figure 19, Figure 20, Figure 21 and Figure 22.

Figure 18 shows that in terms of eigenfrequencies the differences are minimal between the package solution (a) and no-hole limit situation. One can notice that, (c) and (e) are characterized by lower frequencies due to the stiffness reduction of the package induced by the presence of the holes. While, case (d) in Figure 21 shows the higher frequency associated with the sixth mode, because of the modal mass reduction due to the particular shape of the hole. It is worth noting that, during the functioning, the transducer performance involves the vibration of the package walls as well. Hence, the first eigenmode associated with the package walls deformation is reported. It is characterized by the higher eigenfrequency and determines high SPL values in the horizontal direction. The response, also in these cases, was obtained in the frequency domain. All the PMUTs were actuated in parallel with a harmonic voltage excitation of 3 V amplitude and two different bias voltage levels, respectively of 10 V and 3 V, at the corresponding fundamental frequencies of the plates equal to 85.5 kHz (10 V bias) and 93.0 kHz (3 V bias).

Considering the package configurations (a), (b) and (c) at the two analyzed frequencies, the maximum value in the SPL occurs inside each cavity below the transducers. Furthermore, there are high SPL values in the acoustic domain, between the top surface of the die and the protective cap for the case (a) and (b), as the previous no-hole package configuration showed with great reflections of acoustic waves. Regarding the case (c), the presence of a vent directly above each diaphragm let the waves propagate with lower obstructive effect. Consequently, higher acoustic energy is spread in the surrounding air.

**Figure 23:**
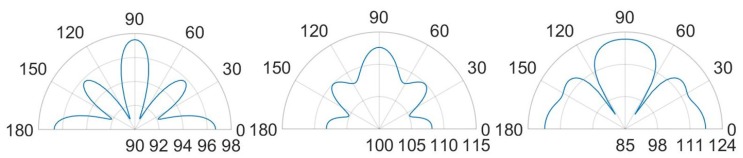
SPL polar plot (dB) at the performing fundamental frequency of 85.5 kHz (10 V bias), at 3.5 cm in the y(90${}^{\circ }$)–z(0${}^{\circ }$) plane: (a) (**left**), (b) (**center**), (c) (**right**).

**Figure 24:**
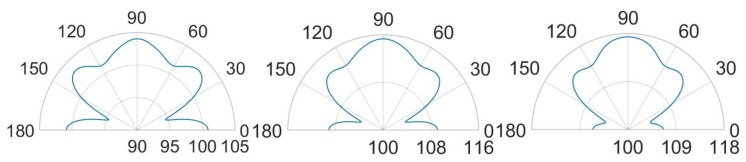
SPL polar plot (dB) at the performing fundamental frequency of 93.0 kHz (3 V bias), at 3.5 cm in the y(90${}^{\circ }$)–z(0${}^{\circ }$) plane: (a) (**left**), (b) (**center**), (c) (**right**).

Further remarkable comments concerning the SPL values and the propagation of the acoustic waves are possible, considering the SPL polar plots, shown in Figure 23 and Figure 24, at a distance of 3.5 cm from the center of the die and in the vertical plane y-z, passing through the center of the system at the analysis frequencies.

In the case (a) at the frequency of 85.5 kHz, Figure 23 reports two narrow lobes around 45${}^{\circ }$ from the vertical acoustic axis of the device together with the vertical and the horizontal ones, related to the involved protective cap eigenmodes of vibration. Hence a strongly directivity is reported with maximum 99.5 dB along the vertical direction, 96 dB at 45–135${}^{\circ }$ and 97 dB at 0–180${}^{\circ }$. In the case (b) at 85.5 kHz, one can notice that higher SPL values and a wider polar plot (Figure 23), due to the fact that there is a larger hole, but the same preferential ways of propagation are still present. Therefore, the maximum is in correspondence of 90${}^{\circ }$ and equal to 112.5 dB, at 45–135${}^{\circ }$ the SPL is 110 dB and at 0–180${}^{\circ }$ it is 108 dB. Regarding the configuration (c) at 85.5 kHz, characterized by the presence of a vent placed above the center of each transducer, the acoustic waves propagate with less reflections inside the air domain between the die and the protective cap, resulting in higher SPL values. In this situation the main vertical lobe, with maximum of 121 dB, is in between the unpackaged and the no-holed package configuration ones (see Figure 16). Moreover, two propagation lobes at around 45${}^{\circ }$ from the vertical axis appear, as well, with local maxima of 115 dB. It is worth noting that a strongly horizontal propagation is reported with SPL value of around 117 dB. As matter of facts, part of the energy excites eigenmodes associated with the vibration of the package walls. The holes placed directly above each transducer reduce the package stiffness and produces a lower obstructive effect for the acoustic propagation.

Actuating the diaphragms at 93.0 kHz, produce wider lobes of propagation with the same shape. In the cases (a) the narrow lobes disappear leaving place to a larger lobe around 90${}^{\circ }$, with maximum value equal to 104 dB, that reaches the two side lobes at 45–135${}^{\circ }$ with local maxima of 103 dB. Furthermore, the horizontal propagation is still present with SPL local maxima equal to 101 dB. The case (b) represents the enlarged situation (a) with maximum SPL of 115 dB along the vertical direction, side lobes at 45–135${}^{\circ }$ with local maxima of 113 dB and horizontal propagation with local maxima of around 109 dB. The case (c) is characterized by a reduced horizontal propagation with local maxima of 106.5 dB. While, the main vertical lobe is present with maximum value equal to 117.5 dB and reaches the two side lobes at 45–135${}^{\circ }$ with local maxima of 114.5 dB.

Considering the package configurations (d) at the two performing frequencies, the hole suggested by the SPL shape on the cap surface, let the acoustic waves follow their natural propagation, generating lower reflections inside the air domain between the protective cap and the die. A lower obstructive effect charactrizes the case (e) due to the presence of several holes. Indeed, as happens in the case (c), greater acoustic energy is spread in the surrounding air with respect the cases (a) and (b). However, the maximum SPL values are reached into the transducers cavities, as well.

**Figure 25:**
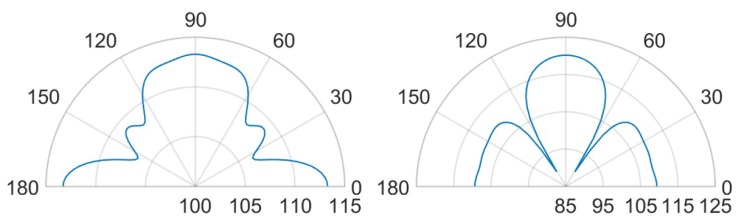
SPL polar plot (dB) at the performing fundamental frequency of 85.5 kHz (10 V bias), at 3.5 cm in the y(90${}^{\circ }$)–z(0${}^{\circ }$) plane: (d) (**left**), (e) (**right**).

**Figure 26:**
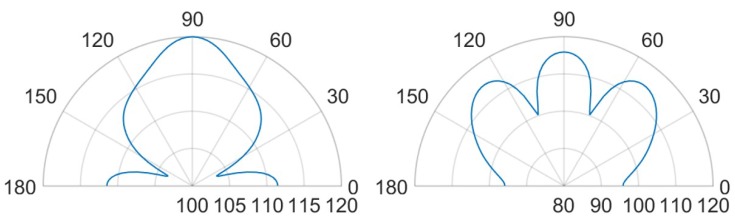
SPL polar plot (dB) at the performing fundamental frequency of 93.0 kHz (3 V bias), at 3.5 cm in the y(90${}^{\circ }$)–z(0${}^{\circ }$) plane: (d) (**left**), (e) (**right**).

Further considerations about the propagation of the acoustic waves and the SPL values are related to the SPL polar plots, shown in Figure 25 and Figure 26, at a distance of 3.5 cm from the center of the die and in the vertical plane y-z, passing through the center of the system at the analysis frequencies.

At the frequency of 85.5 kHz, Figure 25 reports, in the case (d), one wide lobe in the range of 70–110${}^{\circ }$ with local maximum of 113 dB along the vertical direction related to the involved protective cap eigenmodes. Furthermore, the horizontal propagation at 0–180${}^{\circ }$ is due to the package lateral walls vibration with maxima of 113.5 dB. Two narrow specular side lobes around the directions of 40–150${}^{\circ }$ are present with local maxima of 108.5 dB. The case (e), at 85.5 kHz, tends to the case (c), in which there is a coaxial vent directly above each diaphragm, with a wide single vertical lobe with maximum value of 120 dB along the vertical direction and two wide side lobes with local maxima equal to 109 dB along the horizontal direction due to the lateral package walls mode of vibration.

Considering the analysis frequency of 93 kHz, the case (d) shows a SPL polar plot characterized by a wide vertical lobe with maximum value at 90${}^{\circ }$ equal to 120 dB and two narrow horizontal side lobes with local maxima equal to 112 dB. The case (e), at 93 kHz, is characterized by higher directivity due to the presence of a narrower vertical lobe with maximum value equal to 116 dB and two specular side lobes with local maxima of 114 dB at 50–130${}^{\circ }$, related to the protecting cap eigenmodes combination.

### 4.3. SPL Polar Comparison among Different Package Configurations

**Figure 27:**
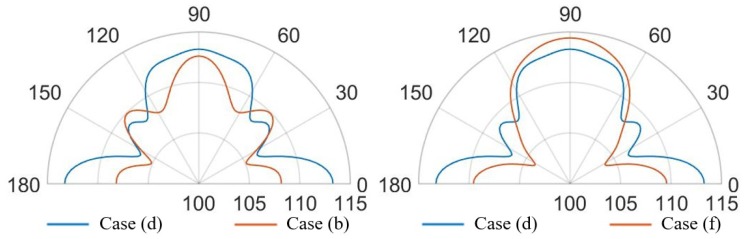
SPL polar plot (dB) at the performing fundamental frequency of 85.5 kHz (10 V bias), at 3.5 cm in the y(90${}^{\circ }$)–z(0${}^{\circ }$) plane: (d) vs (b) (**left**), (d) vs (f) (**right**).

**Figure 28:**
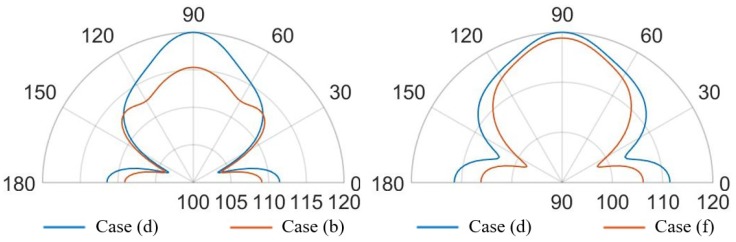
SPL polar plot (dB) at the performing fundamental frequency of 93.0 kHz (3 V bias), at 3.5 cm in the y(90${}^{\circ }$)–z(0${}^{\circ }$) plane: (d) vs (b) (**left**), (d) vs (f) (**right**).

**Figure 29:**
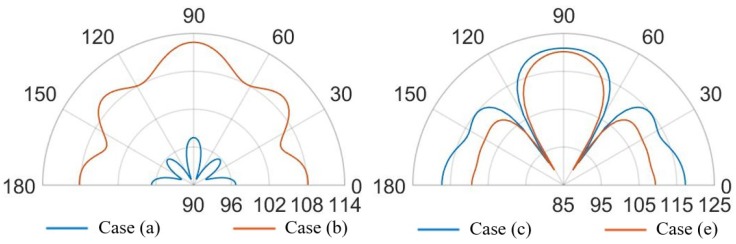
SPL polar plot (dB) at the performing fundamental frequency of 85.5 kHz (10 V bias), at 3.5 cm in the y(90${}^{\circ }$)–z(0${}^{\circ }$) plane: (a) vs (b) (**left**), (c) vs (e) (**right**).

**Figure 30:**
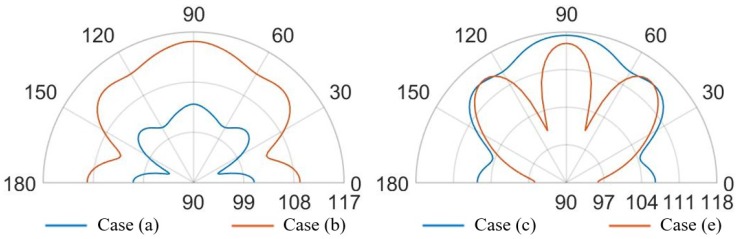
SPL polar plot (dB) at the performing fundamental frequency of 93.0 kHz (3 V bias), at 3.5 cm in the y(90${}^{\circ }$)–z(0${}^{\circ }$) plane: (a) vs (b) (**left**), (c) vs (e) (**right**).

Finally, in Figure 27, Figure 28, Figure 29 and Figure 30 the comparisons of SPL polar plots are reported applying the same scale, at a distance of 3.5 cm from the center of the die and in the vertical plane y-z, passing through the center of the system. To investigate the influence of the SPL shaped hole and compare the packaging solution with the same total holed area $A_{hole}$ of the case (d), a new case (f) characterized by a single circular central hole as case (b) but with a larger hole radius equal to $R_{hole}=\sqrt{A_{hole}/\pi }$, was considered, as well. The numerically compared packaging solutions are: case (d) with SPL shaped hole versus case (b) with protective cap hole of radius $R_{hole}=2R_{PMUT}$ in which the total holed area is four times smaller than the case (d); case (d) versus case (f) with the same total holed area; case (a) with small hole of radius $R_{hole}=R_{PMUT}/3$ versus case (b); case (c) with a coaxial hole placed directly above the center of each transducer versus case (e) with random holes with the same total holed area. Two different analysis frequencies of 85.5 kHz and 93.0 kHz were considered, corresponding to the fundamental performing frequencies of the PMUTs applying respectively 10 V and 3 V bias.

Concerning the comparison between the cases (d) and (b) in Figure 27, the configuration (d) leads to greater propagation in the horizontal direction at 0–180${}^{\circ }$. Hence, with the configuration (d), actuating the system at the fundamental frequency of 85.5 kHz causes the energy excites the eighth eigenmode (see Figure 22), involving the intensively vibration of the package lateral walls as well. Moreover, along the vertical direction the (d) lobe is wider than (b) one with maximum value of 113 dB with respect 112.5 dB and the two symmetrical side lobes are farther from the vertical direction with lower SPL values of about 108.5 dB with respect to 110 dB.

Considering the comparison between the case (d) and (f) with the same holed area, the horizontal propagation is grater in case (d), while along the vertical direction of 90${}^{\circ }$, case (f) shows a higher SPL value of about 114.5 dB and it tends to the situation of no package without any side lobes (see Figure 15). As it can be noticed in Figure 28, exciting the diaphragms at 93.0 kHz, the case (d) shows a main vertical lobe which without any side lobes. The maximum value of SPL is reached at 90${}^{\circ }$ and it is equal to 120 dB, while the horizontal propagation is characterized by the local maximum equal to 112 dB. Concerning the case (b), one can observe that the polar shape is characterized by the main vertical lobe, two side lobes with local maxima at 45–135${}^{\circ }$ and 0–180${}^{\circ }$. The difference in the beam pattern are related to the different eigenmodes involved during the vibration. Furthermore, considering the same holed area of the circular hole at the actuation frequency of 93.0 kHz, the particular lobe shape of the case (f) tends to that one of case (d).

Comparing case (a) and case (b), at the two analysis frequencies, in Figure 29 and Figure 30, is worth noting that the lobe shapes between the two cases are the same. For case (a) the protective cap, with a smaller hole radius then that one of case (b), causes high obstructive effect leading to lower SPL values in every direction. In this two situation the SPL propagation polar plot of (b) encompass that one of case (a). The configuration (b) leads to higher SPL values, reduced and a wider lobe of propagation.

Focusing on Figure 29, the comparison between case (c) and case (e), the shape of the vertical lobe is slightly narrower for case (e) and the intensities are almost the same, but the SPL maximum value are respectively 121 dB and 120 dB. This is due to the presence of the random configuration with a greater number of holes with smaller radii, characterizing the protective cap, which produces a greater obstructive effect than the case (c) in which a larger hole is present directly above each transducer. Concerning Figure 30, the case (e) shows a propagation pattern with three well defined lobes at 90, 45–135${}^{\circ }$ with local maxima of 115 dB, while the case (c) shows a wider polar shape.

## 5. Closing Remarks on the Package Effects

The design of PMUTs should be based on a reliable computational model, that includes the complex coupling among different physics and the interaction among different parts of the device. In this paper, we have considered a 3D multiphysics model that encompasses the mechanical, piezoelectric and acoustic coupling to assess how the package affects the propagation patterns and the acoustic performances of the device considering two the different performing fundamental frequencies of 85.5 kHz (10 V bias) and 93 kHz (3 V bias).

Several simulations were carried out: (i) a geometrically non-linear static analysis to compute the pre-deflected configuration induced by the fabrication residual stresses; (ii) a geometrically non-linear static analysis to compute the starting configuration under the bias voltage account taken the initial stress state; (iii) an electro-mechanical eigenfrequency analysis, around the previously computed starting deformed configuration, due to the prestresses and the bias voltage, to evaluate the eigenfrequencies of the transducers and the vibration eigenmodes of the whole device; (iv) an electro-mechanical-acoustic frequency analysis at 85.5 kHz an 93 kHz, around the starting deflected configuration, under a harmonic voltage perturbation with amplitude of 3 V and a constant bias voltage of 3 V and 10 V.

Several cases were studied and for both the analysis frequencies of 85.5 kHz and 93.0 kHz, related to respectively 10 V and 3 V bias voltages, to evaluate the different package responses at different performing PMUTs fundamental frequencies.

The comparison between the unpackaged and the packaged systems with no vent shows, in the second case, a considerable reduction of SPL and an acoustic propagation essentially governed by the package vibration. While, the package free system propagation pattern is characterized by the spherical waves composition coming from all the vibrating PMUTs.

The protective cap solutions show how the acoustic transmission is strongly influenced by the package presence. This is suggested by the decreasing values of SPL with respect to the unpackaged solution and by the different polar shapes. Nevertheless, the hole presence guarantees higher directivity than the package free case. Therefore, small hole package (a) determines lower SPL values, but induces a narrow lobe in the vertical direction, as it is reported Figure 23. Considering the cap with a central hole of radius equal to $R_{hole}=2R_{PMUT}$, the SPL polar shape is wider and higher value of acoustic intensity are reached (see Figure 23).

Solutions (c) and (e), respectively the case of a hole placed above each transducer and the random holed configuration, are strongly influenced by the analysis frequency. Actuating the vibration at 85.5 kHz induces a propagation pattern with the same shape of unpackaged one, but lower SPL value are reached due to the obstructive effect. While, at 93.0 kHz the acoustic transmission is basically related to the package modes of vibration and the two package cases (c) and (e) differ from the unpackaged one. Finally, the configuration (d) with central hole shaped like the SPL, is particularly efficient to guarantee the wave propagation along the vertical direction, with a wide lobe characterize by high vales of SPL from 60 and 120${}^{\circ }$, at both performing frequencies (see Figure 27 and Figure 28). In this case, the involved vibration of the package walls determines the propagation along the horizontal direction and high SPL values are reached at 0–180${}^{\circ }$, as well.

Future developments will be oriented to the study of different performing frequencies with the applied bias, to evaluate the influence on the acoustic intensity and directivity with respect to the considered package configuration. Furthermore, several analyses will be carried out, varying the distance of the package from the die, the thickness of the package and the hole configuration, to evaluate the effects on the propagation modes and the performances.

## Abbreviations

The following abbreviations are used in this manuscript:

ABC	Absorbing Boundary Condition
ASI	Acoustic-Structure Interaction
EMA	Electro-Mechanical-Acoustic
FSI	Fluid-Structure Interaction
PMUT	Piezoelectric micromachined ultrasonic transducer
SPL	Sound pressure level
